# The CP123L protein of African swine fever virus is a membrane-associated, palmitoylated protein required for viral replication

**DOI:** 10.1128/jvi.01445-24

**Published:** 2024-12-23

**Authors:** Xiangyu Guan, Tao Wang, Yuxuan Gao, Huanjie Zhai, Fengwei Jiang, Qinghe Hou, Xiaoke Yang, Hongxia Wu, Lian-Feng Li, Yuzi Luo, Su Li, Yuan Sun, Hua-Ji Qiu, Yongfeng Li

**Affiliations:** 1 State Key Laboratory for Animal Disease Control and Prevention, CAAS Harbin Veterinary Research Institute 687216 , Harbin, Heilongjiang, China; Lerner Research Institute, Cleveland Clinic, Cleveland, Ohio, USA

**Keywords:** palmitoylation, African swine fever virus, CP123L protein, replication, budding

## Abstract

African swine fever (ASF) is a highly contagious and often lethal disease caused by African swine fever virus (ASFV) in pigs. Protein palmitoylation is a prevalent posttranslational lipid modification that can modulate viral replication. In this study, we investigated the palmitoylation of ASFV proteins. The results revealed that the CP123L protein (pCP123L) of ASFV was palmitoylated at the cysteine residue at position 18 (C18). To further elucidate the functional significance of this posttranslational modification, abolishing palmitoylation through a cysteine-to-serine mutation at C18 (C18S) of pCP123L (pCP123L/C18S) or treatment with 2-bromopalmitate (2-BP), a palmitoylation inhibitor, led to altered cytomembrane localization and migration rate of pCP123L. Furthermore, depalmitoylation achieved through 2-BP treatment significantly suppressed ASFV replication and exerted a profound impact on virus budding. Remarkably, blocking pCP123L palmitoylation *via* the C18S mutation resulted in decreased replication of ASFV. Our study represents the first evidence for the presence of palmitoylation in ASFV proteins and underscores its crucial role in viral replication.

**IMPORTANCE:**

African swine fever (ASF) poses a significant threat to the global pig industry. The causative agent of ASF is African swine fever virus (ASFV), which encodes more than 165 proteins. Protein palmitoylation, a common posttranslational lipid modification, can modulate viral infection. To date, the ASFV proteins that undergo palmitoylation and their impacts on viral replication remain elusive. In this study, the CP123L protein (pCP123L) of ASFV was identified as a palmitoylated protein, and the cysteine residue at position 18 of pCP123L is responsible for its palmitoylation. Notably, our findings demonstrate that palmitoylation plays significant roles in ASFV protein functions and facilitates viral replication.

## INTRODUCTION

Lipidation, a crucial protein modification, encompasses diverse modifications such as *N*-myristoylation, palmitoylation, and farnesylation. Among these lipid modifications, palmitoylation is an extremely prevalent modification, affecting approximately 20% of the cellular proteome (1). At present, palmitoylation has been found in hundreds of mammalian proteins. Protein palmitoylation modulates diverse biological functions of the substrate proteins (2, 3). In mammalian cells, palmitoylation is primarily catalyzed by the palmitoyl transferase (ZDHHC) family. However, due to the low abundance of palmitoylated proteins (4), the difficulty in enriching palmitoylated proteins with high specificity and the high hydrophobicity of intact palmitoyl peptides, relevance, and comprehensive investigation of palmitoylation remain insufficiently explored (5).

Protein palmitoylation can be divided into *S*-palmitoylation, *N*-palmitoylation, and *O*-palmitoylation based on their distinct linkage modes (6, 7). *N*-palmitoylation can occur at certain lysine residues in a protein, where the palmitoyl group is attached to the protein through an amide bond; *O*-palmitoylation involves the linking of the palmitoyl group to the serine residues of the protein *via* ester bonds; *S*-palmitoylation is the modification that covalently binds to the cysteine residues of the protein through sulfur lipid bonds. Palmitoylation is a common and widely studied modification at present. In recent years, more and more studies have found the potential of *S*-palmitoylation as a therapeutic target for anticancer and autoimmune diseases. Most lipid modifications of proteins are irreversible. For example, proteins can undergo dynamic regulation between palmitoylated and depalmitoylated states.

Dynamic palmitoylation exerts a profound influence on protein localization, secretion, stability, and functions by modulating the membrane trafficking process. The reversible nature of *S*-palmitoylation, determined by the structure of the thioester bond linking the acyl and cysteine residues (8, 9), renders it a unique lipid modification that spatiotemporally regulates protein functions. For instance, dynamic fatty acid modification of H-ras facilitates protein trafficking between the cytomembrane and the Golgi apparatus, thereby enabling the changes in subcellular localization for diversified signal transduction. In rats, the synaptic differentiation-induced gene 1 (*SynDIG1*) plays a crucial role in synaptic development. The expression level of *SynDIG1* is decreased upon depalmitoylation (10). Similarly, the palmitoylation of PD-L1 augments its stability, and inhibition of the PD-L1 palmitoylation enhances T-cell immunity against the tumors (11). This suggests that palmitoylation can modulate the stability and distribution of proteins, thereby involving in immune evasion (12). Moreover, *S*-palmitoylation can concurrently fulfill multiple regulatory roles in governing protein functions, while also collaborating with other posttranslational modifications (13). In addition, various palmitoylation inhibitors, such as 2-bromopalmitate (2-BP), of palmitoyl thiolipases have been used *in vitro* and *in vivo* (14).

The impacts of palmitoylation on viral life cycle have been illuminated, with a particular emphasis on its role in facilitating the secretion of infectious particles (14–17). For instance, it has been indicated that palmitoylation determines the subcellular localization, membrane topology, and functions of the hepatitis E virus (HEV) ORF3 protein and plays a pivotal role in the HEV life cycle (14). Moreover, the C-terminal tail of the NS2A protein has been demonstrated to contribute to maintaining Japanese encephalitis virus (JEV) replication efficiency and virulence (16).

African swine fever (ASF) is a highly contagious and often lethal disease of pigs caused by African swine fever virus (ASFV) (18, 19). ASF is classified as a notifiable disease by the World Organization for Animal Health and a Class-A disease in China (20). The ASFV genome ranges from 170 to 194 kb in length and encodes 68 structural proteins and over 100 nonstructural proteins (21). The functions of several ASFV proteins have been well documented (22–26). However, which ASFV proteins are palmitoylated and how they affect viral replication remain completely unknown.

In this study, we identified the palmitoylation of the ASFV CP123L protein (pCP123L) and determined the key cysteine residue that is involved in the palmitoylation. Importantly, our findings demonstrate the significant effects of palmitoylation on ASFV replication and release.

## RESULTS

### The ASFV pCP123L is membrane associated

**Fig 1:**
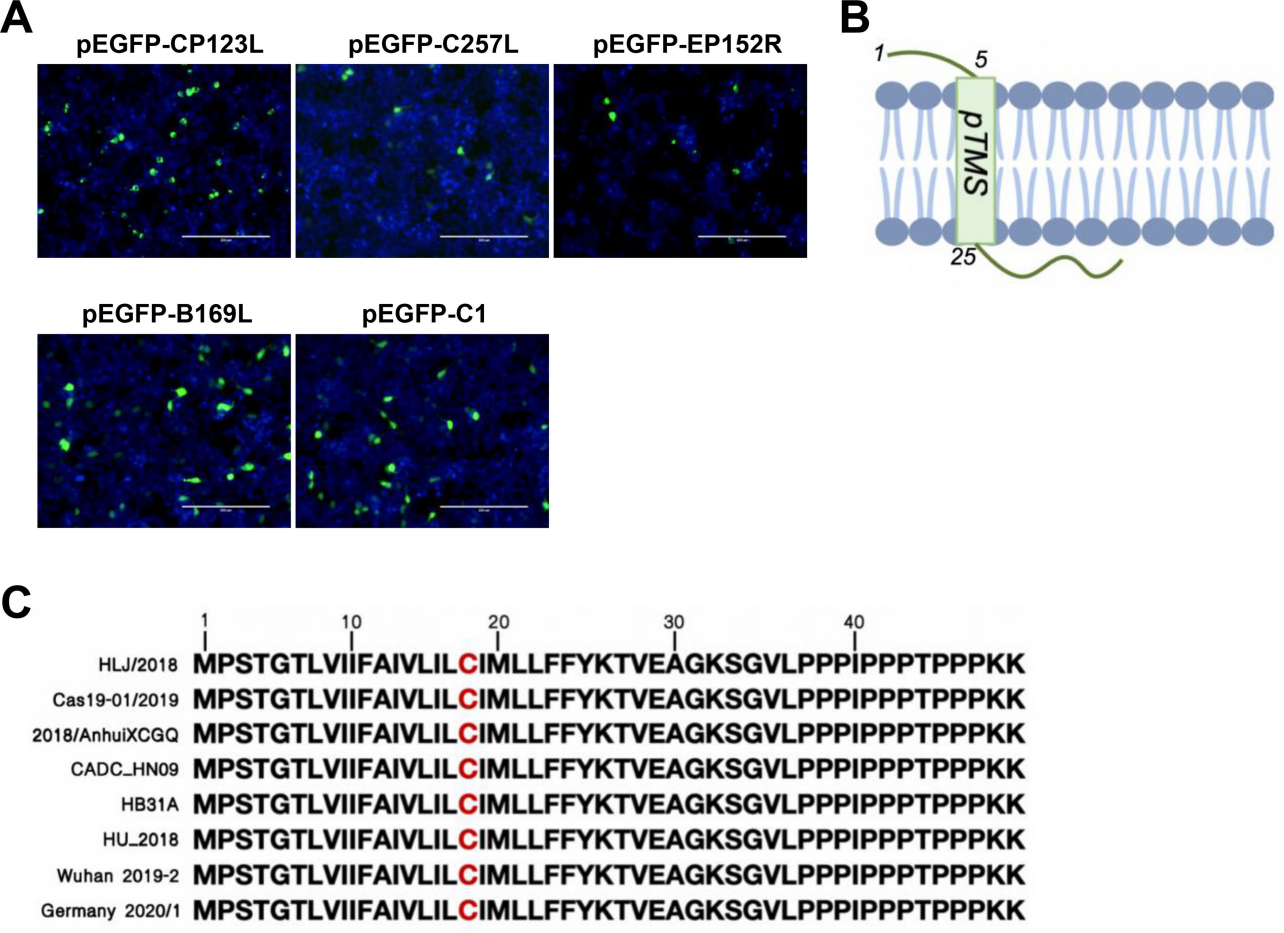
The ASFV pCP123L is membrane-associated. (**A**) Different subcellular localization of the ASFV proteins harboring predicted palmitoylation sites. The plasmids pEGFP-CP123L, pEGFP-EP152R, pEGFP-B169L, pEGFP-C257L, and the control plasmid pEGFP-C1 were transfected into HEK293T cells, and the fluorescence was observed at 48 hours posttransfection. (**B**) The predicted transmembrane region of pCP123L. The top schematic shows the predicted transmembrane region of pCP123L. (**C**) The cysteine presumably responsible for the palmitoylation of pCP123L is highly conserved. Sequence comparison of pCP123L was performed among different ASFV strains.

Fourteen ASFV proteins that may undergo palmitoylation were predicted by bioinformatic features (Table 1), which were selected for further investigation. As demonstrated previously, the process of protein palmitoylation involves the enzymatic attachment of long-chain lipids to the cysteine residues of soluble transmembrane proteins through labile thioester bonds. Based on the fundamental principle above, we performed an initial screening of the ASFV proteins that were predicted to undergo palmitoylation. Initially, the eukaryotic expression plasmids for the selected proteins were constructed and transfected into HEK293T cells. Since it has been shown that protein palmitoylation usually occurs between the cell membrane and the cytoplasmic tail (27–29), pCP123L, pB169L, pEP152R, and pC257L were analyzed first. Nuclear localization was observed for the pEP152R, pB169L, and pC257L proteins. In contrast, pCP123L was found to be localized at the cytomembrane (Fig. 1A). Considering the occurrence of palmitoylation between the membrane and cytoplasmic tail regions, pEP152R, pB169L, and pC257L were excluded from further analysis. To provide evidence supporting the identified palmitoylation, the transmembrane region of pCP123L was predicted (Fig. 1B). Additionally, the cysteine residue presumed to be responsible for the palmitoylation of pCP123L exhibited a high degree of conservation among various ASFV strains (Fig. 1C).

**TABLE 1:** Predicted proteins of ASFV that might undergo palmitoylation

Protein	Transmembrane domain	Palmitoylation site	Function
EP152R	aa 5–24	C8 and C14	Replication (23)
E146L	aa 7–27	C135	Unknown
CP123L	aa 5–25	C18	Unknown
C257L	aa 123–143 and 163–183	C5, C18, C20, and C257	Unknown
B117L	aa 71–91	C14	Permeabilization (24)
B169L	aa 28–48 and 60–80	C19	Unknown
K421R	None	C422	Unknown
E184L	None	C7	Virulence (25)
M448R	None	C98	Protective potential (26)
C122R	None	C4, C7, C10, and C73	Unknown
H124R	None	C24	Unknown
C717R	None	C94, C95, and C96	Unknown
H240R	None	C23	Stabilizing capsid (30)
F317L	None	C177	Unknown

### The ASFV pCP123L is palmitoylated at residue C18

**Fig 2:**
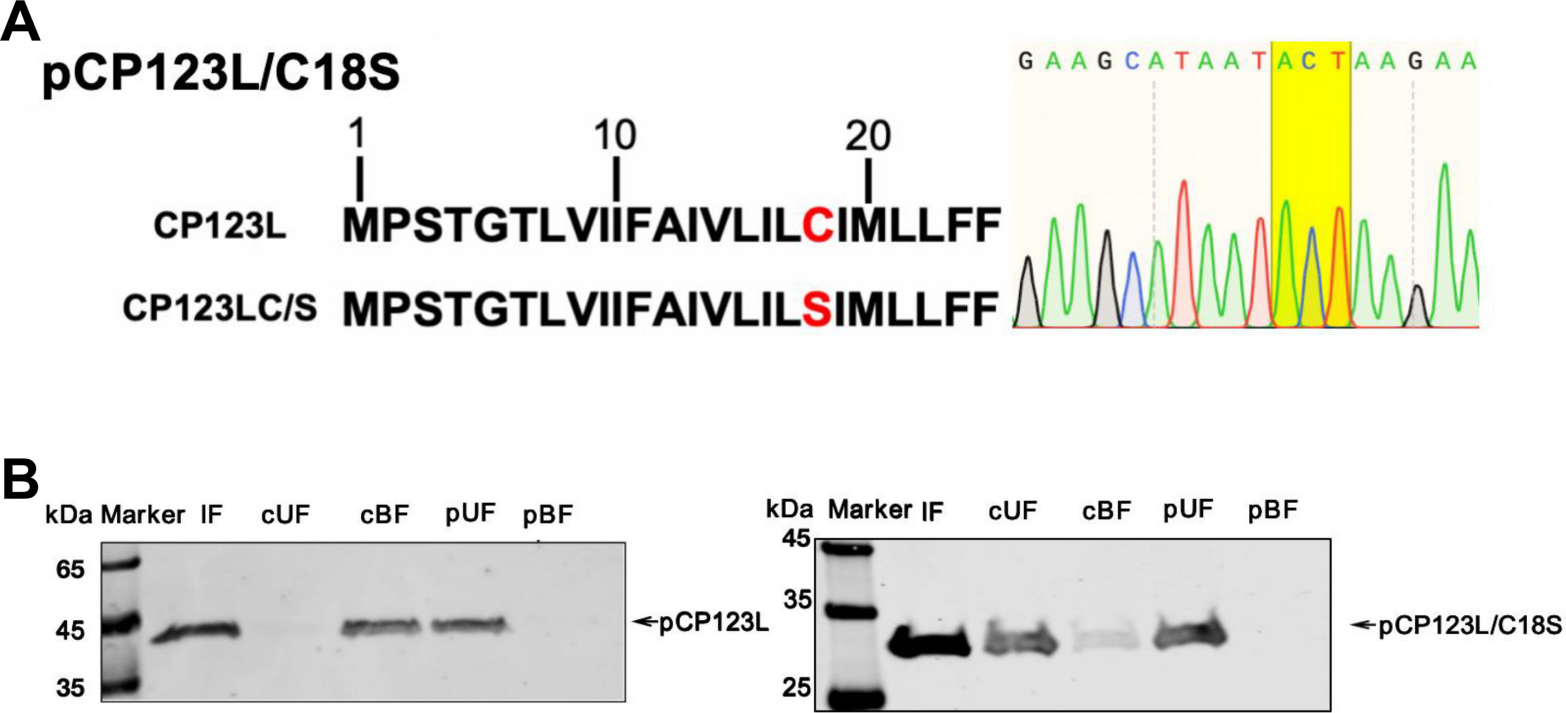
The ASFV pCP123L is palmitoylated at the residue C18. (**A**) Sequence analysis of pCP123L/C18S. The palmitoylation site is shown in red. Site-directed mutagenesis at the predicted cysteine site was performed by replacing the cysteine with serine. (**B**) Identification of the palmitoylation status of pCP123L and pCP123L/C18S. The *S*-palmitoylation of the ASFV pCP123L was examined using a palmitoylation kit. The proteins were examined by the Badrilla’s CAPTUREome *S*-palmitoylated protein kit and immunoblotted using an antibody specific for palmitoylated proteins. Lane 1, input fraction (IF) post-block; Lane 2, cleaved unbound fraction (cUF), after treatment with the thioester cleavage reagent; Lane 3, cleaved bound fraction (cBF), proteins recovered from the resin after treatment with the thioester cleavage reagent; Lane 4, preserved unbound fraction (pUF), after treatment with the acyl preservation reagent; Lane 5, preserved bound fraction (pBF), proteins recovered from the resin after treatment with the acyl preservation reagent.

Next, the palmitoylation status of the ASFV pCP123L was examined using a palmitoylation kit. First, according to the predicted palmitoylation sites, a plasmid expressing the pCP123L mutant pCP123L/C18S (cysteine-to-serine mutation at position 18 of pCP123L) was constructed (Fig. 2A). In contrast to pCP123L/C18S, pCP123L was depleted from the thioester cleavage reagent treatment. The cleaved unbound fraction (cUF) (Lane 2) was quantitatively recovered in the cleaved bound fraction (cBF) (Lane 3), indicating the presence of the *S*-palmitoylation in pCP123L (Fig. 2B). The data demonstrate that pCP123L is palmitoylated at the C18 residue.

### The subcellular localization and electrophoretic mobility of the ASFV pCP123L are associated with palmitoylation

**Fig 3:**
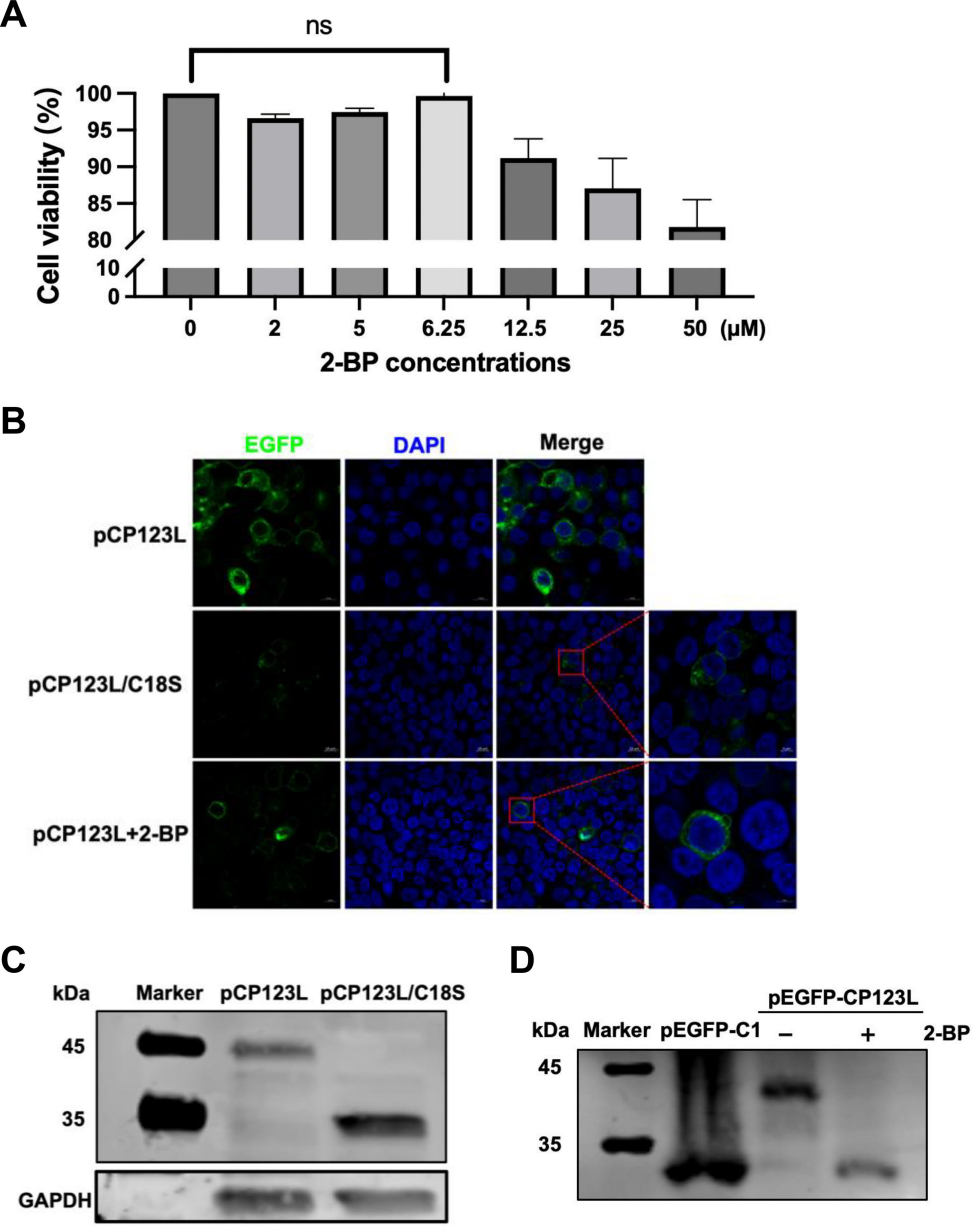
Palmitoylation alters the subcellular localization and electrophoretic mobility of the ASFV pCP123L. (**A**) Cytotoxicity of 2-bromopalmitate (2-BP) as a palmitoylation inhibitor to HEK293T cells. The cytotoxicity of 2-BP to HEK293T cells was tested by using the cell viability assay kit. (**B**) Distinct subcellular localization of pCP123L and its mutant. HEK293T cells were transfected with the plasmid pEGFP-CP123L expressing pCP123L or the plasmid pEGFP-CP123L/C18S expressing the mutant pCP123L. The fluorescence of the cells was observed by laser confocal microscopy at 48 hours posttransfection (hpt). (**C**) The changes in the electrophoretic mobility of pCP123L by mutating the cysteine responsible for the palmitoylation. HEK293T cells were transfected with the recombinant plasmid pEGFP-CP123L or the mutant plasmid pEGFP-CP123L/C18S. At 48 hpt, the proteins were lysed and collected for Western blotting. (**D**) Altered electrophoretic mobility of pCP123L following treatment with 2-BP. HEK293T cells were transfected with the plasmid pEGFP-CP123L or the control plasmid pEGFP-C1 and subsequently subjected to treatment with 2-BP or left untreated for 24 hours. The samples were subjected to Western blotting analysis.

First, the impacts of palmitoylation on the subcellular localization of the ASFV pCP123L were examined. The effects of 2-BP on cell viability were evaluated. The results showed that 2-BP did not affect the cell viability at 6.25 µM concentration (Fig. 3A). The HEK293T cells transfected with the plasmid expressing pCP123L were treated with 2-BP of various concentrations, while the subcellular localization of pCP123L and the corresponding mutant pCP123L/C18S was examined by laser confocal microscopy. The results revealed that pCP123L was localized at the cell membrane and cytoplasm with a punctate distribution, whereas the punctate distribution of pCP123L/C18S nearly vanished. The subcellular localization of pCP123L/C18S in the HEK293T cells remained consistent with that treated with the palmitoylation inhibitor (Fig. 3B). Collectively, palmitoylation modulates the intracellular distribution of the ASFV pCP123L.

To investigate the impacts of palmitoylation on the electrophoretic mobility of pCP123L, HEK293T cells were transfected with the plasmids encoding pCP123L or its corresponding mutant pCP123L/C18S. At 48 hours posttransfection (hpt), the lysates were collected for Western blotting. The data indicated that the mobility of pCP123L was changed to some extent after depalmitoylation (Fig. 3C), suggesting that palmitoylation affects the mobility of the ASFV pCP123L. Alternatively, depalmitoylation could result in accelerated migration of pCP123L. To further validate the impact of palmitoylation on protein migration, protein samples treated with 2-BP were lysed at 48 hpt to obtain total cellular proteins for subsequent Western blotting analysis. As depicted in Fig. 3D, pCP123L exhibited altered molecular weight upon treatment with 2-BP. Collectively, our findings support the conclusion that palmitoylation influences the migration rate of the ASFV pCP123L.

### Depalmitoylation inhibits the replication of the reporter ASFV

**Fig 4:**
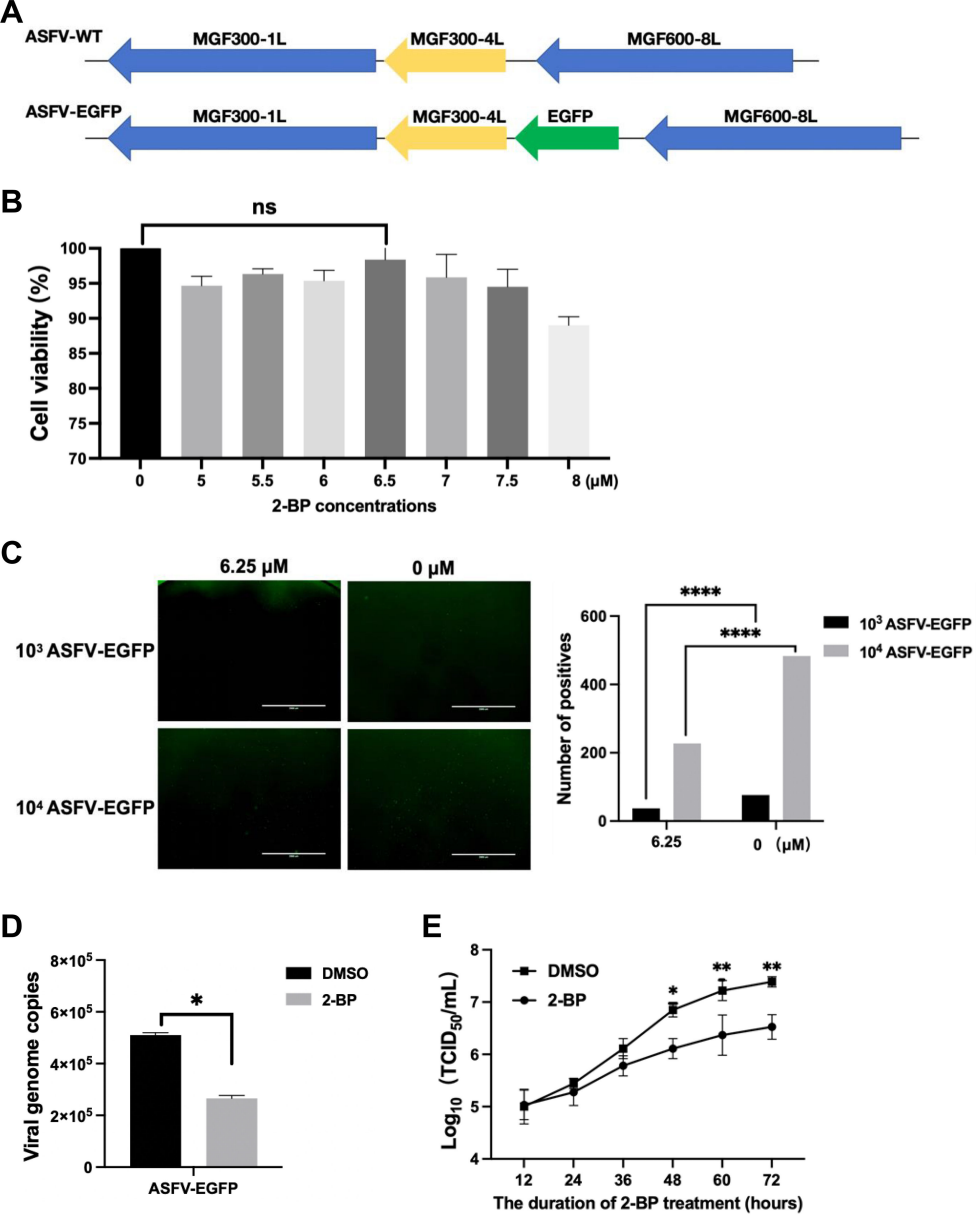
Depalmitoylation suppresses the replication of the reporter ASFV. (A) Construction diagram illustrating the reporter ASFV-EGFP carrying the *EGFP* gene inserted downstream of the *MGF300-4L* gene. (B) Effects of 2-bromopalmitate (2-BP) as a palmitoylation inhibitor on cell viability for PAMs. PAMs were treated with the indicated concentrations of 2-BP and subsequently evaluated for cell viability using the CCK-8 kit. (C to E) Depalmitoylation inhibits the replication of the reporter ASFV. PAMs were infected with ASFV-EGFP at a multiplicity of infection of 0.1 for 24 hours, followed by treatment with 2-BP or dimethyl sulfoxide (DMSO) serving as a negative control for another 24 hours. The number of fluorescent cells was counted, the virus genome copies were tested by qPCR, and the supernatants were titrated at various time points to determine the viral titers expressed as TCID_50_.

To investigate the effects of palmitoylation on ASFV replication, a reporter ASFV (ASFV-EGFP) was constructed by inserting the *EGFP* gene downstream the *MGF300-4L* gene (Fig. 4A). Various concentrations of 2-BP were added to the reporter ASFV-infected primary porcine alveolar macrophages (PAMs), and fluorescence was observed at 72 hours postinfection (hpi). The effects of the inhibitor on cell viability were assessed, which indicated that 6.25 µM 2-BP did not affect the cell viability (Fig. 4B). The results demonstrated that 2-BP significantly decreased the number of the ASFV-infected fluorescent cells (Fig. 4C), the genome copies (Fig. 4D), and the viral titers (Fig. 4E) of the ASFV-EGFP in comparison with the treatment with dimethyl sulfoxide (DMSO) serving as a negative control.

### Depalmitoylation impairs the replication of wild-type ASFV

**Fig 5:**
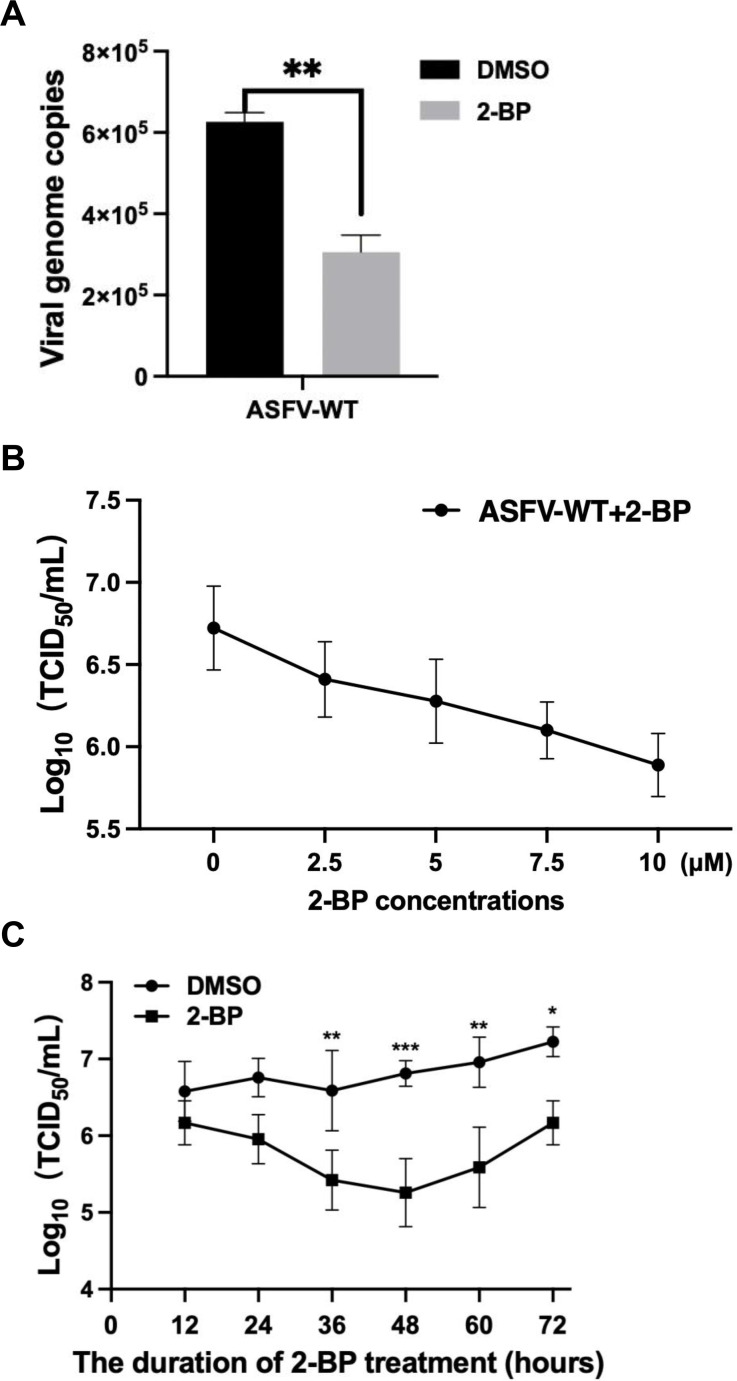
Depalmitoylation effectively impedes the replication of the wild-type ASFV. (**A**) Depalmitoylation inhibits the replication of the wild-type ASFV (ASFV-WT). PAMs were infected with ASFV-WT at an MOI of 0.1 and treated with 2-bromopalmitate (2-BP) as a palmitoylation inhibitor or dimethyl sulfoxide (DMSO) as a negative control for 24 hours. After the cells were freeze-thawed twice, the supernatants were collected to extract DNA, and the virus genome copies were tested by qPCR. (**B**) Depalmitoylation exerts an inhibitory effect on ASFV infection in a dose-dependent manner. PAMs were infected with ASFV and treated with 2-BP of 0, 2.5, 5, 7.5, or 10 µM for 24 hours. The virus was harvested at 48 hours postinfection, and the viral titers and its growth kinetics were determined by indirect immunofluorescence assay. (**C**) Depalmitoylation significantly suppresses ASFV replication. PAMs were infected with ASFV-WT for the indicated times and treated with 2-BP or DMSO for 24 hours. The ASFV-infected cells were collected at different time points to determine the viral titers as TCID_50_.

Next, the effects of palmitoylation on the wild-type ASFV HLJ/18 strain (ASFV-WT) replication were further investigated. The results revealed a significant decrease in genome copies following 2-BP treatment (Fig. 5A). The impacts of palmitoylation on the titers of ASFV-WT were subsequently analyzed. The PAMs infected with ASFV-WT were treated with varying concentrations of 2-BP for 24 hours. Subsequently, the samples were collected at 48 hpi and subjected to examination by indirect immunofluorescence assay (IFA). The results demonstrated a dose-dependent inhibition of ASFV replication upon treatment with 2-BP (Fig. 5B).

Furthermore, PAMs were infected with ASFV-WT and treated with 2-BP for 24 hours. Subsequently, the samples were collected at 12, 24, 36, 48, 60, and 72 hpi and subjected to examination by IFA. It was observed that the treatment of the inhibitor significantly decreased the titers of ASFV, particularly after a period of 36 hours (Fig. 5C). Collectively, depalmitoylation suppresses ASFV replication.

### Palmitoylation affects the release of wild-type ASFV

**Fig 6:**
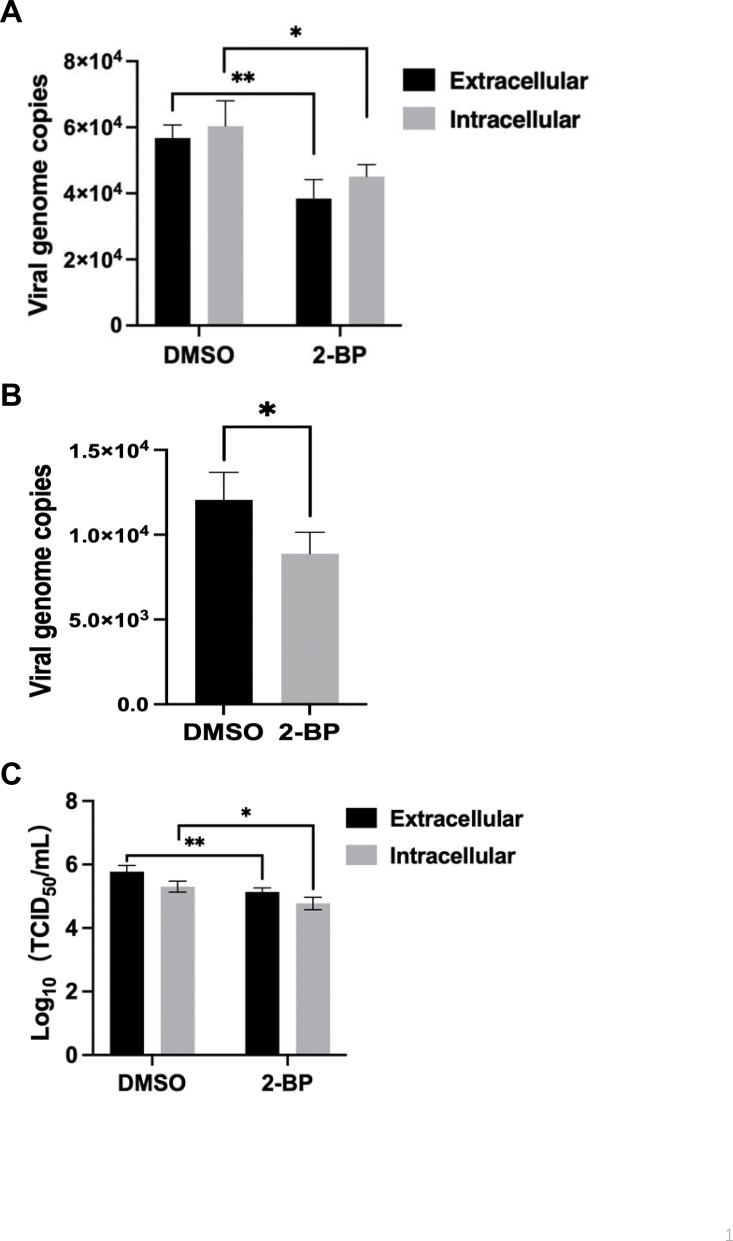
Palmitoylation is involved in the release of ASFV. (**A**) No significant difference in the viral genome copies between intracellular and extracellular compartments. PAMs were infected with the wild-type ASFV (ASFV-WT) for 24 hours and treated with 2-bromopalmitate (2-BP) as a palmitoylation inhibitor or dimethyl sulfoxide (DMSO) as a negative control for 24 hours. The virus samples from both intracellular and extracellular compartments of the cells were harvested to determine the viral genome copies. (**B**) Effects of palmitoylation on ASFV attachment. The genomic copies of the bound virions in the PAMs infected with ASFV-WT were quantified by qPCR. (**C**) Palmitoylation is involved in the release of ASFV. PAMs were infected with ASFV for 24 hours, followed by treatment with 2-BP or DMSO for 24 hours, and the titers of intracellular and extracellular viruses were quantified by indirect immunofluorescence assay.

Previous studies have demonstrated that palmitoylation plays a role in viral assembly and secretion (14–17, 31). To investigate the impacts of palmitoylation on ASFV assembly and secretion, PAMs were infected with ASFV-WT for 24 hours, followed by treatment with 2-BP for 24 hours. The results demonstrated no difference in the extracellular or intracellular viral genome copies between the 2-BP-treated and the DMSO-treated groups (Fig. 6A).

To investigate the impacts of palmitoylation on ASFV binding, PAMs were incubated with ASFV-WT at 4°C for 2 hours. The binding viral particles were quantified using real-time PCR (qPCR). Remarkably, the treatment with 2-BP resulted in a significant reduction in ASFV genome copies (Fig. 6B).

Functional consequences were assessed by the determination of the infectivity in the intracellular and extracellular compartments for the changes following depalmitoylation. While a similar infectivity was observed intracellularly for both constructs, the budding of ASFV was significantly impaired upon 2-BP treatment (Fig. 6C). These results indicate that the cysteine residue of the ASFV protein engaged in palmitoylation is essential for the secretion of infectious particles.

### The palmitoylation **at the C18** of the ASFV pCP123L contributes to ASFV replication

**Fig 7:**
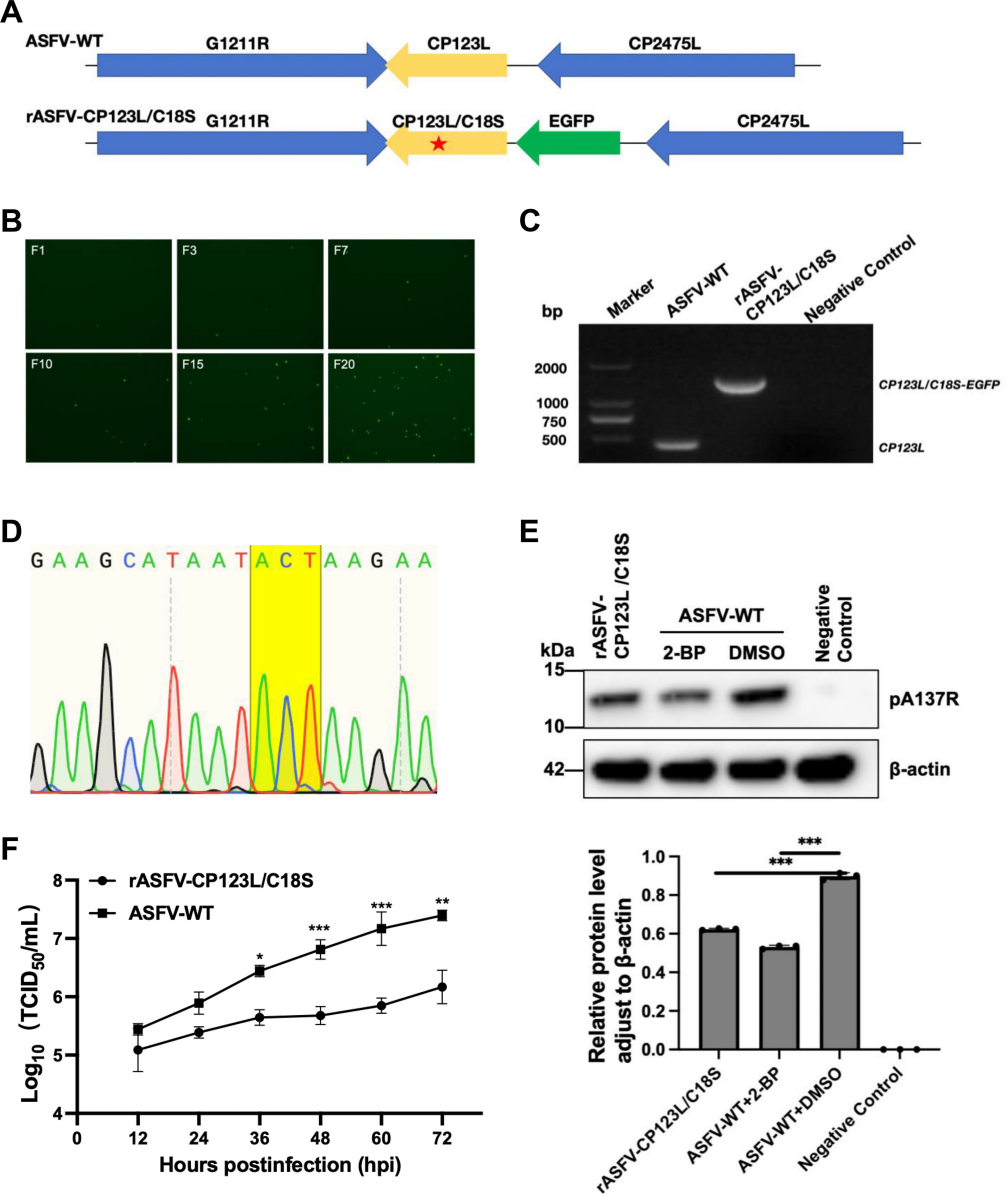
Depalmitoylation of pCP123L results in decreased replication of ASFV. (**A**) Schematic diagram of the recombinant virus rASFV-CP123L/C18S. The ASFV mutant harbored the C18S mutation in the *CP123L* gene, and the *EGFP* gene was inserted upstream of the *CP123L* gene in the ASFV genome. (**B**) Purification of the recombinant virus. PAMs were transfected with the transfer vector pOK12-CP123L/C18S and infected with the wild-type ASFV (ASFV-WT), and the recombinant virus was purified by multiple rounds of limiting dilutions. (**C**) Identification of the *CP123L* gene mutation of rASFV-CP123L/C18S. The *CP123L* gene of ASFV-WT (Lane 1) or the fusion gene *CP123L*/*C18S-EGFP* of rASFV-CP123L/C18S (Lane 2) was amplified by PCR using the primers ASFV-CP123L/C18S-123-F/ASFV-CP123L/C18S-M-R. (**D**) Sequence analysis of the *CP123L* gene of the recombinant virus. (**E**) The expression of the A137R protein (pA137R) in the PAMs infected with rASFV-CP123L/C18S is decreased in contrast to that of ASFV-WT. PAMs were infected with ASFV-WT or rASFV-CP123L/C18S at an MOI of 3 and harvested at 48 hours postinfection for analyzing the pA137R expression by Western blotting using anti-pA137R polyclonal antibodies. The intensities of the protein bands were determined by the ImageJ software. The relative expression level of pA137R was normalized to that of *β*-actin. (**F**) Growth curve of rASFV-CP123L/C18S.

To further investigate the effects of palmitoylation of the cysteine residue at position 18 of pCP123L on ASFV infection, we generated a depalmitoylated ASFV mutant, designated as rASFV-CP123L/C18S (Fig. 7A). Briefly, *CP123L/C18S* was fused with *EGFP* under the control of the *p72* promoter and inserted into the pOK-12 vector, creating the transfer vector pOK12-CP123L/C18S. PAMs were transfected with pOK12-CP123L/C18S, followed by infection with ASFV-WT. Finally, the mutant virus was purified through limiting dilution based on the fluorescence observation (Fig. 7B).

To further evaluate the purification of the recombinant virus, viral DNA was extracted from the infected cells and determined by PCR using the primers targeting the *CP123L/C18S-EGFP* gene (Table 2). The *CP123L/C18S-EGFP* gene was amplified from the genomic DNA extracted from the cells infected with rASFV-CP123L/C18S, but not with ASFV-WT, while the *CP123L* gene was detected from ASFV-WT, but not from rASFV-CP123L/C18S (Fig. 7C), indicating the generation of the recombinant virus. Sequence analysis revealed the insertion of the *CP123L/C18S-EGFP* fusion gene in the expected locus (Fig. 7D). Except for the mutation of the cassette, no undesired mutations in the rASFV-CP123L/C18S genome were observed during the process of purification, as evidenced by the next-generation sequencing.

**TABLE 2:** Primers used in this study

Primer	Sequence (5’−3’)
pEGFP-CP123L-F	GTACAAGTCCGGACTCAGATCTCGAGGCCACCATGCCCTCGACTGGGACACT
pEGFP-CP123L-R	GCGGTACCGTCGACTGCAGAATTCTTAATACTTCTTAAGAAACTCTT
pEGFP-E146L-F	GTACAAGTCCGGACTCAGATCTCGAGGCCACCATGGGCGGCACTACAGACTT
pEGFP-E146L-R	GCGGTACCGTCGACTGCAGAATTCTTAAATAATACGCTGTAGTCCGG
pEGFP-EP152R-F	GTACAAGTCCGGACTCAGATCTCGAGGCCACCATGTACTCCATTCTCATTGC
pEGFP-EP152-R	GCGGTACCGTCGACTGCAGAATTCTTAATTTTGTGTTATATATTTTT
pEGFP-B169L-F	GTACAAGTCCGGACTCAGATCTCGAGGCCACCATGAATGTAGATTTTATTGC
pEGFP-B169L-R	GCGGTACCGTCGACTGCAGAATTCTTAATTATTATATTGAGAAGGCA
pEGFP-C257L-F	GTACAAGTCCGGACTCAGATCTCGAGGCCACCATGTACTCCGTCTGCGATGT
pEGFP-C257L-R	GCGGTACCGTCGACTGCAGAATTCTTAACAATTAGGGCCGGGAGGCA
pEGFP-CP123L/C18S-F	GTACAAGTCCGGACTCAGATCTCGAGGCCACCATGCCCTCGACTGGGACACT
pEGFP-CP123L/C18S-R	GCGGTACCGTCGACTGCAGAATTCTTAATACTTCTTAAGAAACTCTT
ASFV-CP123L/C18S-L-F	GGTACCCGGGAGCTCGAATTCCCCCTCTTATTGAAATCGTT
ASFV-CP123L/C18S-L-R	TTTAAAGAGTTTCTTAAGAAGTATTAGGAAATAAAGTCGTAGCA
ASFV-CP123L/C18S-123-F	TGCTACGACTTTATTTCCTAATACTTCTTAAGAAACTCTTTAAA
ASFV-CP123L/C18S-123-R	CATCTCTCACGAGATCGTGACATGCCCTCGACTGGGAC
ASFV-CP123L/C18S-M-F	GTCCCAGTCGAGGGCATGTCACGATCTCGTGAGAGATG
ASFV-CP123L/C18S-M-R	AAACGTTACTTATATAACAAATCCTGTGAGATCATGGCAGCT
ASFV-CP123L/C18S-R-F	AGCTGCCATGATCTCACAGGATTTGTTATATAAGTAACGTTT
ASFV-CP123L/C18S-R-R	GTCTGCAGAAGCTTCGAATTCGAGTTACTATCCAAAGGCAAC

Subsequently, a comparative analysis of viral protein expression was performed between rASFV-CP123L/C18S and ASFV-WT by Western blotting. The results demonstrated a decreased expression of the A137R protein in the PAMs infected with rASFV-CP123L/C18S compared with those infected with ASFV-WT at 48 hpi (Fig. 7E).

Finally, the replication dynamics of of rASFV-CP123L/C18S was evaluated by the multi-step growth curve analysis. The virus was harvested at various time points for IFA. The results demonstrated that the replication of rASFV-CP123L/C18S exhibited a substantial decrease in contrast to ASFV-WT (Fig. 7F). Collectively, the palmitoylation of the ASFV pCP123L at C18 contributes to the efficient replication of ASFV *in vitro*.

## DISCUSSION

In the present study, we identified the cysteine of the ASFV pCP23L as the determinant for membrane association. More precisely, we found that pCP123L is palmitoylated at the *N*-terminal cysteine. The palmitoylation of the ASFV pCP123L determines its membrane association and subcellular localization. Moreover, the cysteine residue of pCP123L was engaged in palmitoylation, which significantly affects the release of infectious ASFV.

The bioinformatics approach greatly facilitates the prediction of candidate palmitoylated ASFV proteins. In this study, bioinformatics analysis was employed to predict the palmitoylation status of ASFV target proteins, and the results suggested that pCP123L, pB169L, pEP152R, and pC257L from ASFV may be palmitoylated. Given that palmitoylation predominantly occurs at the interface between the cell membrane and the cytoplasmic tail (32, 33), a comprehensive analysis was conducted to determine the subcellular localization of the predicted ASFV proteins. Both fluorescence observation and transmembrane prediction indicate that pCP123L was membrane-associated. Importantly, the palmitoylation status of the ASFV pCP123L was identified using a palmitoylation kit, and the results showed that the protein was palmitoylated.

Many studies have shown significant alterations in the subcellular localization of proteins upon depalmitoylation (17, 34, 35). It has been demonstrated that palmitoylation regulates the subcellular localization of Lck to the cytomembrane. Following depalmitoylation, the double-site mutants exhibited impaired cytomembrane targeting and subsequent downstream signaling activation in T cells. This suggests that depalmitoylation alters the subcellular localization of Lck (34). In addition, it has also been found that palmitoylation can stabilize protein membrane localization (17, 35). In this study, the involvement of palmitoylation in the subcellular localization of pCP123L was investigated, and the results showed that the subcellular localization of pCP123L was altered after depalmitoylation.

It has been confirmed that the palmitoylation is essential for the viral assembly, budding, and infectivity (14–17, 31, 36–41). While palmitoylation of the viral proteins can contribute to viral replication, palmitoylation of antiviral proteins is important for inhibiting viral infection (13, 39). However, the precise mechanisms underlying the involvement of protein palmitoylation in viral infection remain elusive (40). It has been shown that depalmitoylation can inhibit the secretion of infectious particles and that the infectivity of viruses is compromised to a certain extent after depalmitoylation, suggesting the crucial significance of palmitoylation in virus assembly and infectivity (41). In the present study, the effects of palmitoylation on ASFV replication and ASFV budding were examined. The results showed that depalmitoylation inhibits ASFV replication and budding following treatment with 2-BP. Therefore, it is intriguing to ascertain whether the palmitoylation at C18 of CP123L is involved in ASFV infection, which will benefit the understanding of the significance of the palmitoylation of pCP123L in ASFV infection. By using the recombinant virus rASFV-CP123L/C18S harboring the CP123L/C18S mutation, it was observed that abolishing palmitoylation through the C18S mutation resulted in the reduced replication of ASFV in PAMs. These data suggest that the pCP123L palmitoylation at C18 contributes to the replication efficiency of ASFV. Based on these findings, we speculate that the palmitoylation of pCP123L might be associated with the pathogenicity of ASFV in pigs, which warrants further investigation in the future.

Although progress has been made in the understanding of the mechanism underlying protein palmitoylation, many physiological functions remain to be defined (5). The precise modification sites and exact functional roles of various palmitoylated proteins necessitate further investigation. Only about 100 palmitoylated proteins have been identified from yeast to mammals, with unclear functions and substrates for various palmitoyl acyltransferases (PATs) (42–48). Despite the advances in understanding the protein palmitoylation through site-directed mutagenesis and inhibition assays, the research process remains time-consuming, labor-intensive, and challenging to identify modification sites without prior information. This is primarily due to several reasons. First, the enzymology of *S*-palmitoylation and depalmitoylation of proteins is still largely unknown, despite the identification of some conserved DHHC motifs in cysteine-rich domains for certain protein acyltransferases and deacylases (49). Second, there is no consensus motif for protein palmitoylation modification. Palmitoylation predominantly occurs in transmembrane or membrane-associated proteins with low solubility and abundance (50). Third, their hydrophobicity increases with the addition of long-chain fatty acids, further complicating their detection compared with other posttranslational modifications like phosphorylation and glycosylation, which benefit from affinity groups, such as specific antibodies enriching low-abundance palmitoylated proteins. While PATs mediate protein palmitoylation (51), the underlying mechanism remains unclear. We need to continue our efforts in qualitative functional exploration and quantitative analysis of the palmitoylated proteins using more effective and accurate research methods, which will undoubtedly help deepen our understanding of the mechanisms underlying protein palmitoylation, enrich proteomics knowledge, promote the development of proteomic studies on protein palmitoylation, and hold profound implications for the diagnosis, treatment, and tumor therapy for animal or human diseases.

In summary, palmitoylation regulates the subcellular localization and dynamic mobility of the ASFV pCP123L, which is involved in viral replication.

## MATERIALS AND METHODS

### Cells, viruses, and antibodies

The ASFV HLJ/18 strain (GenBank accession no. MK333180.1) was isolated from field samples in China (52), and the reporter ASFV-EGFP was constructed in the background of ASFV-WT. PAMs and HEK293T cells were cultured with RPMI 1640 medium (catalog no. C11875500BT; Gibco) supplemented with 10% heat-inactivated fetal bovine serum (FBS) (catalog no. 10099–141C; Gibco) and 4% antibiotics–antimycotics (10,000  IU/mL penicillin, 10,000  µg/mL streptomycin, and 25  µg/mL amphotericin B) (catalog no. 15240–062; Gibco) in a 37°C incubator with 5% CO_2_. A mouse anti-p72 monoclonal antibody (MAb) was purchased from Ingenasa (catalog no. 160620; Ingenasa). Anti-A137R polyclonal antibodies were homemade (53).

### Construction of plasmids

The ASFV *CP123L*, *EP152R*, *B169L*, and *C257L* genes were amplified by PCR and cloned into the pEGFP-C1 vector using the primers listed in Table 2, creating the recombinant plasmids pEGFP-CP123L, pEGFP-EP152R, pEGFP-B169L, and pEGFP-C257L, respectively.

### Palmitoylation analysis

For each sample, 1–2 mg of the total cellular proteins was thoroughly mixed with 500 µL of a blocking buffer, which comprised 24 µL of a thiol-blocking reagent (catalog no. K010-311B, Badrilla), diluted in 3 mL of buffer A (catalog no. K010-311A, Badrilla). After vortexing for 5 seconds, the samples were incubated at 40°C for 4 hours with constant shaking. Three volumes (1.5 mL) of ice-cold acetone were added to each sample, followed by vortexing for 5 seconds and precipitating at –20°C for 20 minutes. The protein pellet was recovered by centrifuging at 16,000 × *g* for 5 minutes, followed by discarding the supernatant. The pellet was washed five times with 1 mL of ice-cold 70% acetone for 5 seconds each time and then centrifuged at 16,000 × *g* for 1 minute. The CAPTUREome capture resin (catalog no. K010-311F, Badrilla) was prepared by adding 2 mL of 1 × binding buffer to one tube (catalog no. K010-311F, Badrilla) and incubating on a rotary wheel for 30 minutes. The washed resin was centrifuged at 16,000 × *g* for 1 minute. Next, the supernatants were aspirated, and the resin was resuspended in 275 µL of 1 × binding buffer at a ratio of settled resin to buffer of 1:1.

After vortexing for 5 seconds, 50 µL of the resin slurry was added to each sample. Simultaneously, the thioester cleavage reagent was prepared by combining 200 µL of neutralization buffer (catalog no. K010-311H, Badrilla) with one vial of thioester cleavage reagent (catalog no. K010-311D, Badrilla). Prior to use, the thioester cleavage reagent was vortexed for 5 seconds and then left undisturbed for 1 minute to ensure complete dissolution. Subsequently, 19 µL of the thioester cleavage reagent solution was added to each experimental sample while adding 19 µL of the acyl preservation reagent (catalog no. K010-311E, Badrilla) to each negative control sample. The reaction proceeded at room temperature with constant agitation for 2.5 hours. The samples were centrifuged at 16,000 × *g* for 1 minute, and the supernatant was removed, leaving only 50 µL. Then, 50 µL of 2× Laemmli sample buffer (catalog no. K010-311G, Badrilla) was added. For experimental samples, this fraction was referred to as cUF. For negative control samples, this fraction was designated as preserved unbound fraction (pUF).

The resin was washed five times with 1 mL of 1 × binding buffer mixer and was centrifuged for 1 minute at 16,000 × *g* each time to recover the resin. After the final wash, the captured proteins were eluted from the resin using 50 µL of 2 × Laemmli sample buffer (catalog no. K010-311G, Badrilla). We thus obtained cBF in the experimental samples and the preserved bound fraction (pBF) in the negative control samples.

### Western blotting

The recombinant plasmids were transfected into HEK293T cells using the HP transfection regent (catalog no. 06366236001, Roche). The cells were lysed in a buffer containing 1% NP-40 and 1 mM phenylmethylsulfonyl fluoride at 48 hpt. Immunoblotting was performed after SDS-PAGE according to a standard protocol. Briefly, the proteins were transferred onto the Immobilon-P membranes (Millipore, Bedford, MA, USA), and the membranes were blocked with phosphate-buffered saline (PBS) containing 3% skimmed cow milk and 5% bovine serum albumin (BSA) for 2 hours at 20°C. The blots were incubated for 1 hour with 5 mL of mouse anti-EGFP MAb (1:1,000), followed by washing three times with PBS containing 0.1% Tween 20. The IRDye 800CW goat anti-mouse IgG (H + L) was used at a dilution of 1:5,000 for detecting the bound primary antibody.

PAMs were infected with rASFV-CP123L/C18S or ASFV-WT at a multiplicity of infection (MOI) of 3 and harvested at 48 hpi for determining the A137R protein expression using anti-pA137R polyclonal antibodies and the IRDye 800CW goat anti-rabbit IgG (H + L). Following three washes with PBST, the target proteins were scanned using the Odyssey Infrared Imaging System (Li-Cor BioSciences, USA). Finally, the intensities of the bands were quantified by the ImageJ software.

### Confocal microscopy

HEK293T cells were cultured on glass-bottom cell culture dishes with a specific diameter. The cells were then fixed in 4% paraformaldehyde for 20 minutes. After permeabilization with 0.2% Triton X-100 for 15 minutes, the cells were washed three times with PBS, followed by additional incubation with DAPI (ThermoFisher) to allow staining of the nuclei. The cells were examined under a Zeiss LSM 980 Quasar laser scanning fluorescence confocal microscope, and images were analyzed with the ImageJ software.

### Cytotoxicity assay

PAMs or HEK293T cells (100 µL/well) were seeded in 96-well plates and cultured overnight in an incubator. Different concentrations of 2-BP as a palmitoylation inhibitor were added to the culture plate and incubated for 24 hours, followed by addition of 10 µL of CCK-8 solution to each well. The plates were incubated in the incubator for 4 hours. The absorbance at OD_450nm_ was determined by a microplate reader.

### qPCR

The ASFV genome was extracted from the cells or cell supernatants using the MagaBio plus virus DNA purification kit (catalog no. 9109; BioFlux) according to the manufacturer’s protocols. The ASFV genome copies were quantified by qPCR on the QuantStudio system (Applied Biosystems, USA) based on a previously described method.

### IFA

PAMs seeded in 96-well plates at a density of 80% were infected with ASFV strains. After culturing for 48 hours, the cells were washed twice with sterile PBS. Next, each well was fixed with pre-cooled absolute ethanol (–20°C) for 30 minutes and then rinsed twice with PBS. Anti-p72 MAb (diluted at 1:200) was added as the primary antibody (100 µL per well) and incubated at 37°C for 2 hours. After washing three times with PBS, a secondary antibody consisting of FITC-labeled goat anti-mouse antibody (diluted at 1:300; 100 µL per well) was then added and incubated in the dark at 37°C for 1 hour. Following three additional washes, the fluorescence was observed and recorded using an inverted fluorescence microscope.

### Virus titration

PAMs were infected with the virus at an MOI of 0.1, followed by fixation in 4% paraformaldehyde for 20 minutes. Subsequently, the cells underwent permeabilization with 0.2% Triton X-100 for 15 minutes and were rinsed three times with PBS. The 50% tissue culture infective dose (TCID_50_) of the virus was determined by IFA.

### Virus assembly and budding assays

PAMs were inoculated with ASFV and treated with 2-BP for 24 hours. At 48 hpi, the samples were collected from both intracellular and extracellular compartments to determine viral titers and genome copies.

### Virus adsorption assay

Virus adsorption assay was conducted in PAMs grown in 24-well plates. For the binding assay, the cells were infected with equal numbers of equal titers of virions for 2 hours at 4°C. After washing five times with PBS, the cells were lysed in the buffer for DNA extraction and examination of the ASFV genome copies. All the samples were analyzed in triplicates.

### Generation of an ASFV mutant harboring depalmitoylated pCP123L

To generate the transfer vector pOK12-CP123L/C18S, the corresponding fusion genes were amplified by overlapping PCR with the primers listed in Table 2. The resulting plasmid was transfected into PAMs, followed by ASFV infection. The recombinant ASFV was purified by limiting dilution based on the fluorescent intensities, as described previously (25, 30).

### Next-generation sequencing of the rASFV-CP123L/C18S genome

The PAMs seeded in 6-well plates were infected with rASFV-CP123L/C18S, followed by DNA extraction, as described above. The full-length genome was sequenced utilizing the MGISEQ-200 platform and aligned with the genome of the ASFV HLJ/18 strain using SnapGene.

### Statistical analyses

Significance values were calculated by using the unpaired *t* test with the GraphPad Prism 6 software package. Differences were considered significant if the *P* value was < 0.05.

## Data Availability

All the data presented in this study are available. The reads covering the recombinant ASFV genome generated by the next-generation sequencing were aligned with the genome of the ASFV HLJ/18 strain (GenBank accession no. MK333180.1). The raw sequencing reads are available upon request.
